# Dipotassium hydrogencarbonate fluoride monohydrate

**DOI:** 10.1107/S1600536813006041

**Published:** 2013-03-09

**Authors:** Volker Kahlenberg, Timo Schwaier

**Affiliations:** a University of Innsbruck, Institute of Mineralogy & Petrography, Innrain 52, A-6020 Innsbruck, Austria

## Abstract

Single crystals of the title compound, K_2_(HCO_3_)F·H_2_O, were obtained as a secondary product after performing flux synthesis experiments aimed at the preparation of potassium rare earth silicates. The basic building unit of the structure is an [(HCO_3_)(H_2_O)F]^2−^ zigzag chain running parallel to [001]. Both types of anions as well as the water mol­ecules reside on mirror planes perpendicular to [010] at *y* = 0.25 and *y* = 0.75, respectively. Linkage between the different constituents of the chains is provided by O—H⋯O and O—H⋯F hydrogen bonding. The K^+^ cations are located between the chains and are coordinated by two F and five O atoms in form of a distorted monocapped trigonal prism.

## Related literature

For phase equilibria in the system (Na,K)–(CO_3_,HCO_3_,F)–H_2_O, see: Soliev & Nizimov (2009 ▶, 2011 ▶, 2012 ▶). For structure determinations of phases in the system K–(CO_3_,HCO_3_,F)–H_2_O, see: Arlt & Jansen (1990 ▶); Beurskens & Jeffrey (1964 ▶); Broch *et al.* (1929 ▶); Cirpus & Adam (1995 ▶); Hill & Miller (1927 ▶); Preisinger *et al.* (1994 ▶); Skakle *et al.* (2001 ▶); Thomas *et al.* (1974 ▶). For phases related to the title compound, see: Dinnebier & Jansen (2008 ▶); Margraf *et al.* (2003 ▶); Pritchard & Islam (2003 ▶). For bond-valence parameters, see: Brown & Altermatt (1985 ▶). For details of the synthetic procedure, see: Vidican *et al.* (2003 ▶).

## Experimental

### 

#### Crystal data

K_2_(HCO_3_)F·H_2_O
*M* *_r_* = 176.24
Monoclinic, 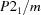

*a* = 5.4228 (4) Å
*b* = 7.1572 (6) Å
*c* = 7.4539 (7) Å
β = 105.121 (9)°
*V* = 279.28 (4) Å^3^
*Z* = 2
Mo *K*α radiation
μ = 1.64 mm^−1^
*T* = 173 K
0.18 × 0.18 × 0.06 mm

#### Data collection

Agilent Xcalibur (Ruby, Gemini ultra) diffractometer
Absorption correction: multi-scan (*CrysAlis PRO*; Agilent, 2011 ▶) *T*_min_ = 0.946, *T*_max_ = 1
1008 measured reflections
551 independent reflections
475 reflections with *I* > 2σ(*I*)
*R*_int_ = 0.027

#### Refinement

*R*[*F* ^2^ > 2σ(*F* ^2^)] = 0.028
*wR*(*F* ^2^) = 0.075
*S* = 1.05
551 reflections
52 parameters
4 restraints
All H-atom parameters refined
Δρ_max_ = 0.31 e Å^−3^
Δρ_min_ = −0.31 e Å^−3^

### 

Data collection: *CrysAlis PRO* (Agilent, 2011 ▶); cell refinement: *CrysAlis PRO*; data reduction: *CrysAlis PRO*; program(s) used to solve structure: *SIR2002* (Burla *et al.*, 2003 ▶); program(s) used to refine structure: *SHELXL97* (Sheldrick, 2008 ▶); molecular graphics: *ATOMS for Windows* (Dowty, 2011 ▶); software used to prepare material for publication: *publCIF* (Westrip, 2010 ▶);.

## Supplementary Material

Crystal structure: contains datablock(s) global, I. DOI: 10.1107/S1600536813006041/wm2728sup1.cif

Structure factors: contains datablock(s) I. DOI: 10.1107/S1600536813006041/wm2728Isup2.hkl

Additional supplementary materials:  crystallographic information; 3D view; checkCIF report

## Figures and Tables

**Table 1:** Hydrogen-bond geometry (Å, °)

*D*—H⋯*A*	*D*—H	H⋯*A*	*D*⋯*A*	*D*—H⋯*A*
O4—H41⋯O1^i^	0.84 (2)	1.89 (2)	2.723 (3)	176 (3)
O4—H42⋯F^ii^	0.84 (2)	1.87 (2)	2.707 (3)	177 (3)
O3—H3⋯F^iii^	0.81 (2)	1.72 (2)	2.529 (3)	174 (4)

Symmetry codes: (i) 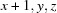; (ii) 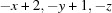; (iii) 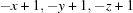.

**Fig. 1.**
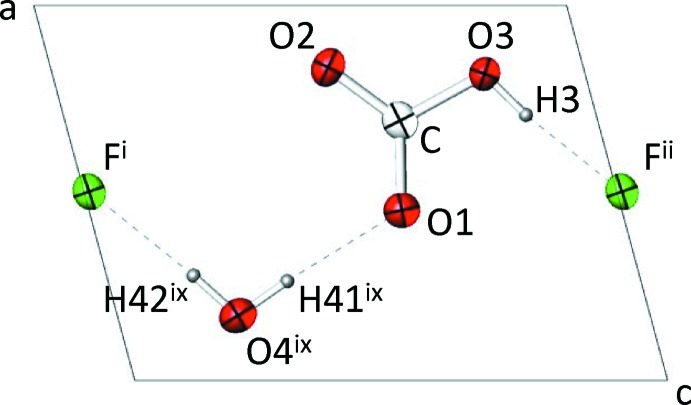
Projection of a single [(HCO3)(H2O)F]2- – chain in y = 0.25 parallel to [010]. C—O and O—H bonds are given as thick and thin open lines, respectively. Dashed lines correspond to H···O and H···F hydrogen bonds; thermal ellipsoids have been drawn on the 70% probability level. [Symmetry codes: (i) -x + 1, -y + 1, -z; (ii) -x + 1, -y + 1, -z + 1; (ix) x - 1, y, z].

**Fig. 2.**
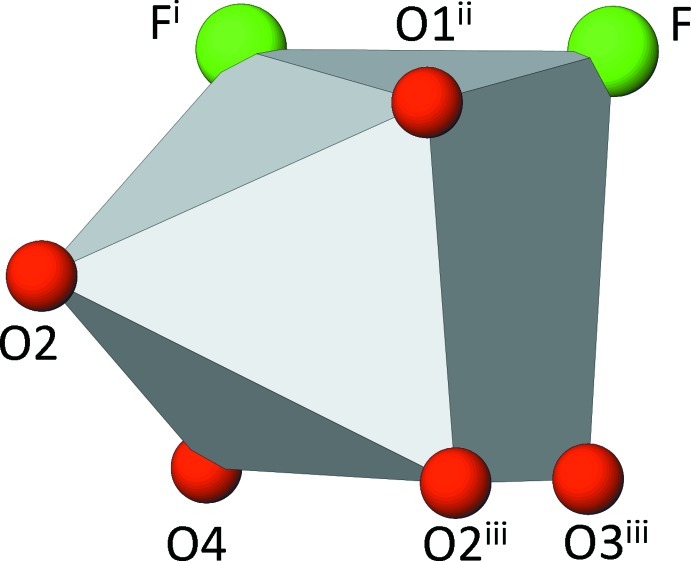
Side view of the coordination polyhedron around a single K+ cation. [Symmetry codes: (i) -x + 1, -y + 1, -z; (ii) -x + 1, -y + 1, -z + 1; (iii) -x + 2, -y + 1, -z +1].

**Fig. 3.**
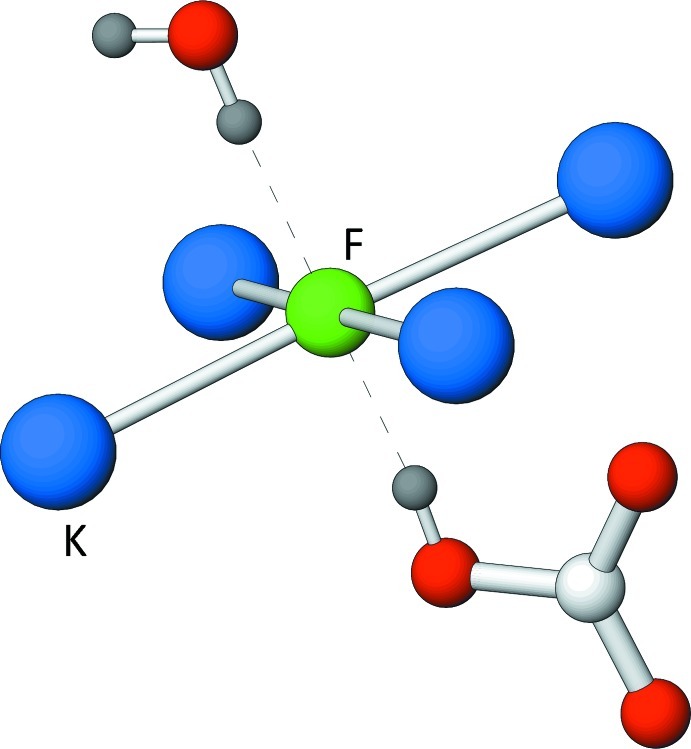
Coordination environment around a single F- anion.

**Fig. 4.**
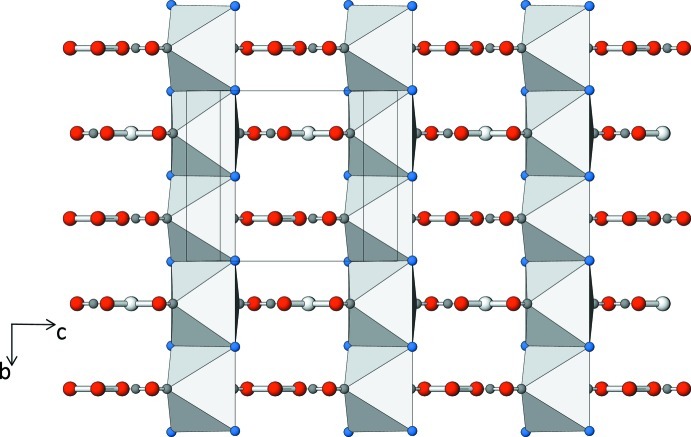
Projection of edge-sharing anion-centered chains (linked by H2O and HCO3 – moieties) perpendicular to (100). Small blue spheres correspond to the K+ cations.

**Fig. 5.**
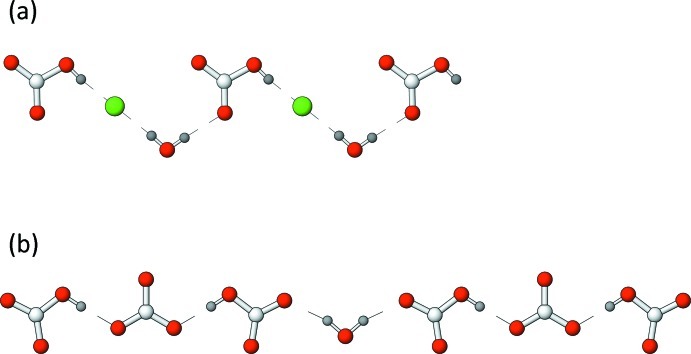
Chains containing carbonate/hydrogencarbonate and water moieties in (a) K2(HCO3)F.H2O and (b) (NH4)4[H2(CO3)3].H2O.

**Fig. 6.**
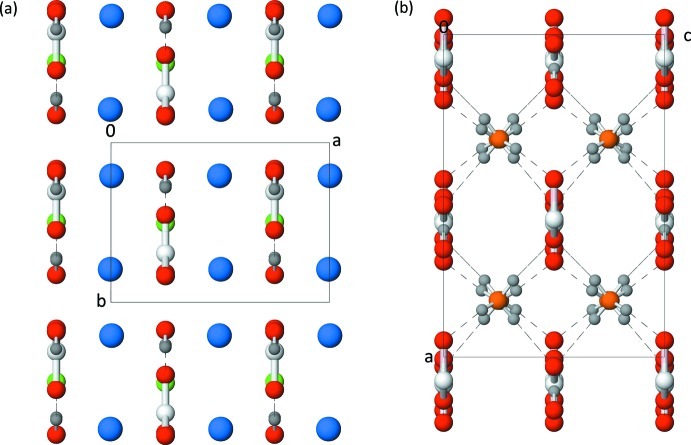
Projections of the crystal structures of (a) K2(HCO3)F.H2O and (b) (NH4)4[H2(CO3)3].H2O parallel to the chain directions. Small blue spheres correspond to the K+ cations.

## supplementary crystallographic information

### Comment

In the present paper we describe a previously unknown phase within the multinary system K–(CO_3_,HCO_3_,F)–H_2_O which represents the first example of an alkali metal double salt containing hydrogencarbonate as well as fluoride anions.

Basic building units of the structure are [(HCO_3_)(H_2_O)F]^2-^-zigzag-chains running along the [001]-direction. Within the unit cell the chains are located on mirror planes at *y* = 0.25 and *y* = 0.75, respectively. As can be seen from Fig. 1, a single water molecule in the chain provides two intermolecular hydrogen bonds with neighboring hydrogencarbonate and fluorine anions. Each fluorine atom in turn is the acceptor of two hydrogen bonds from adjacent H_2_O and (HCO_3_)-moieties (Table 1). The C—O bond lengths (1.250 (3)–1.350 (4) Å) and the O—C—O bond angles (114.7 (2)–127.6 (3) °) are in meaningful ranges. The distortions of the hydrogencarbonate group (point group symmetry *m*) follow the expected trends: the considerably longer C—O3 bond represents the linkage between the central carbon atom and the OH-group, which is also involved in hydrogen bonding. Consequently, the O1—C—O3 and O2—C—O3 angles are significantly smaller than 120°, whereas the O1—C—O2 angle is larger compared with the ideal undistorted case.

The potassium cations are coordinated by two fluorine and five oxygen atoms. The resulting coordination polyhedron can be described as a distorted monocapped trigonal prism (Fig. 2). Bond valence sum calculations were performed for the K^+^ cations using the bond valence parameters for K—O and K—F of Brown & Altermatt (1985). The resulting value in valence units (v.u.) is 1.10 and thus is reasonably close to the expected value of 1.0 v.u..

A slightly different description of the structure results when anion-centred polyhedra are considered as well. Actually, each F^-^ anion is surrounded by two hydrogen atoms (belonging to a water molecule and a hydrogencarbonate group, respectively) and four additional equatorial potassium atoms in form of a distorted octahedron (Fig. 3). Edge-sharing between adjacent octahedra results in columns running along [010]. The linkage between the octahedral columns with the laterally attached H_2_O and (HCO_3_)-groups is realised by O4—H41··· O1 hydrogen bonds (Fig. 4).

To our best knowledge, the present compound represents the first example of an alkali hydrogencarbonate halogenide hydrate. The structure of an alkaline earth hydrogencarbonate chloride hydrate with composition Mg(HCO_3_)_3_Cl.6H_2_O has been reported recently (Dinnebier & Jansen, 2008). However, there are no closer structural relationships between the two phases. On the other hand, anionic chains containing (CO_3_)/(HCO_3_)-ions and water molecules have been observed in several other hydrous alkali/ammonium carbonates and hydrogencarbonates such as Na_2_CO_3_.H_2_O (Pritchard & Islam, 2003), K_2_CO_3_.1.5H_2_O (Skakle *et al.*, 2001) and K_4_H_2_(CO_3_)_3_.1.5H_2_O (Cirpus & Adam, 1995). Concerning the general shape of the chains, the present compound and (NH_4_)_4_[H_2_(CO_3_)_3_].H_2_O (Margraf *et al.*, 2003) are closely related (Fig. 5). As can be seen from Fig. 6, in both compounds the chains are located on mirror planes.

Only recently, the phase equilibria in the multinary system (Na,K)—(CO_3_,HCO_3_,F)—H_2_O have been studied at different temperatures in the range between 273 and 323 K (Soliev & Nizimov, 2009, 2011, 2012). The authors verified the existence of the following equilibrium solid phases containing potassium, for all of which detailed structural characterizations have been already performed in previous investigations: K_2_CO_3_.1.5H_2_O (Skakle *et al.*, 2001), KHCO_3_ (Thomas *et al.*, 1974), 2KHCO_3_.K_2_CO_3_.1.5H_2_O (or K_4_H_2_(CO_3_)_3_.1.5 H_2_O) (Cirpus & Adam, 1995) and KF (Broch *et al.*, 1929). On the other hand, potassium carbonate hexahydrate (K_2_CO_3_.6H_2_O) has been identified as the stable hydrate below about 267 K (Hill & Miller, 1927). For potassium fluoride two hydrated forms have been described: KF.2H_2_O (Preisinger *et al.*, 1994) and KF.4H_2_O (Beurskens & Jeffrey, 1964). Furthermore, an anhydrous potassium fluoro-carbonate with composition K_3_F(CO_3_) was reported (Arlt & Jansen, 1990).

### Experimental

Single crystals of K_2_(HCO_3_)F.H_2_O were obtained by chance as a by-product during the preparation of K-*REE*-silicates (*REE* is an rare earth element) from a KF flux following the approach of Vidican *et al.* (2003). After the removal of the platinum crucible the solidified melt cake was immediately crashed in an agate mortar and transferred to a glass slide under a polarizing binocular. Due to the hygroscopic character of potassium fluoride under the given experimental conditions (temperature: 296 K, relative humidity: 43%) deliquescence of the halide became obvious after 30 min. After 2 h, 50 µm sized platy birefringent single crystals were observed in the solution droplets which continued to grow until the solution was completely evaporated. The sample was checked regularly over a period of two weeks. However, no indication for weathering or alteration could be detected. In order to study the compound in more detail a single-crystal of good optical quality showing sharp extinction when imaged between crossed polarizers was selected and mounted on the tip of a glass fibre using nail hardener as glue. A preliminary unit cell determination at ambient temperature using on Oxford Diffraction Gemini Ultra single-crystal diffractometer resulted in a set of lattice parameters that could not be found in the recent WEB-based version of the Inorganic Crystal Structure Database (ICSD). Therefore, we decided to perform a full data collection for structure solution. For the low temperature measurement the crystal was flash-cooled in a 173 (2) K dried air stream generated by an Oxford Cryosystems Desktop Cooler.

### Refinement

Difference Fourier calculations revealed the positions of all missing hydrogen atoms. For the subsequent refinement the positional parameters of the H atoms were optimized with restraints using *DFIX* 0.84 (2) commands for the O—H distances and *DFIX* 1.32 (2) commands for the H···H distances (giving H—O—H angles close to 105°).

### Crystal data

**Table:**

K_2_(HCO_3_)F·H_2_O	*F*(000) = 176
*M**_r_* = 176.24	standard setting
Monoclinic, *P*2_1_/*m*	*D*_x_ = 2.096 Mg m^−^^3^
Hall symbol: -P 2yb	Mo *K*α radiation, λ = 0.71073 Å
*a* = 5.4228 (4) Å	Cell parameters from 538 reflections
*b* = 7.1572 (6) Å	θ = 2.8–28.5°
*c* = 7.4539 (7) Å	µ = 1.64 mm^−^^1^
β = 105.121 (9)°	*T* = 173 K
*V* = 279.28 (4) Å^3^	Plate, colourless
*Z* = 2	0.18 × 0.18 × 0.06 mm

### Data collection

**Table:**

Agilent Xcalibur (Ruby, Gemini ultra) diffractometer	551 independent reflections
Radiation source: Enhance (Mo) X-ray Source	475 reflections with *I* > 2σ(*I*)
Graphite monochromator	*R*_int_ = 0.027
Detector resolution: 10.3575 pixels mm^-1^	θ_max_ = 25.3°, θ_min_ = 2.8°
ω scans	*h* = −5→6
Absorption correction: multi-scan (*CrysAlis PRO*; Agilent, 2011)	*k* = −8→7
*T*_min_ = 0.946, *T*_max_ = 1	*l* = −8→7
1008 measured reflections	

### Refinement

**Table:**

Refinement on *F*^2^	Primary atom site location: structure-invariant direct methods
Least-squares matrix: full	Secondary atom site location: difference Fourier map
*R*[*F*^2^ > 2σ(*F*^2^)] = 0.028	Hydrogen site location: inferred from neighbouring sites
*wR*(*F*^2^) = 0.075	All H-atom parameters refined
*S* = 1.05	*w* = 1/[σ^2^(*F*_o_^2^) + (0.0329*P*)^2^] where *P* = (*F*_o_^2^ + 2*F*_c_^2^)/3
551 reflections	(Δ/σ)_max_ < 0.001
52 parameters	Δρ_max_ = 0.31 e Å^−^^3^
4 restraints	Δρ_min_ = −0.31 e Å^−^^3^

### Special details

**Table:**

Geometry. All s.u.'s (except the s.u. in the dihedral angle between two l.s. planes) are estimated using the full covariance matrix. The cell s.u.'s are taken into account individually in the estimation of s.u.'s in distances, angles and torsion angles; correlations between s.u.'s in cell parameters are only used when they are defined by crystal symmetry. An approximate (isotropic) treatment of cell s.u.'s is used for estimating s.u.'s involving l.s. planes.
Refinement. Refinement of *F*^2^ against ALL reflections. The weighted *R*-factor *wR* and goodness of fit *S* are based on *F*^2^, conventional *R*-factors *R* are based on *F*, with *F* set to zero for negative *F*^2^. The threshold expression of *F*^2^ > 2σ(*F*^2^) is used only for calculating *R*-factors(gt) *etc*. and is not relevant to the choice of reflections for refinement. *R*-factors based on *F*^2^ are statistically about twice as large as those based on *F*, and *R*- factors based on ALL data will be even larger.

### Fractional atomic coordinates and isotropic or equivalent isotropic displacement parameters (Å^2^)

**Table:**

	*x*	*y*	*z*	*U*_iso_*/*U*_eq_	
K	0.79241 (9)	0.50302 (5)	0.23529 (7)	0.0170 (2)	
F	0.4966 (3)	0.75	−0.0096 (2)	0.0164 (5)	
O4	1.1731 (4)	0.25	0.2263 (3)	0.0231 (6)	
H41	1.265 (6)	0.25	0.336 (2)	0.028*	
H42	1.280 (5)	0.25	0.163 (4)	0.028*	
O1	0.4539 (4)	0.25	0.5871 (3)	0.0212 (6)	
O2	0.8344 (4)	0.25	0.5210 (3)	0.0186 (5)	
O3	0.8172 (4)	0.25	0.8112 (3)	0.0182 (5)	
H3	0.708 (5)	0.25	0.868 (4)	0.022*	
C	0.6926 (6)	0.25	0.6289 (4)	0.0150 (7)	

### Atomic displacement parameters (Å^2^)

**Table:**

	*U*^11^	*U*^22^	*U*^33^	*U*^12^	*U*^13^	*U*^23^
K	0.0181 (3)	0.0121 (3)	0.0193 (4)	−0.00008 (16)	0.0021 (3)	−0.00045 (18)
F	0.0177 (10)	0.0170 (10)	0.0140 (10)	0	0.0034 (8)	0
O4	0.0165 (12)	0.0340 (14)	0.0198 (13)	0	0.0065 (9)	0
O1	0.0168 (12)	0.0290 (12)	0.0179 (12)	0	0.0046 (9)	0
O2	0.0198 (11)	0.0205 (11)	0.0171 (12)	0	0.0079 (9)	0
O3	0.0153 (11)	0.0262 (12)	0.0136 (12)	0	0.0049 (9)	0
C	0.0195 (17)	0.0082 (14)	0.0168 (17)	0	0.0037 (14)	0

### Geometric parameters (Å, º)

**Table:**

K—F^i^	2.6816 (11)	O4—H42	0.838 (17)
K—F	2.7389 (11)	O1—C	1.250 (3)
K—O1^ii^	2.7541 (17)	O1—K^vii^	2.7541 (17)
K—O2	2.7590 (17)	O1—K^ii^	2.7541 (17)
K—O4	2.7599 (17)	O2—C	1.250 (4)
K—O3^iii^	2.8477 (18)	O2—K^vi^	2.7590 (17)
K—O2^iii^	2.9330 (16)	O2—K^iii^	2.9330 (16)
F—K^i^	2.6816 (11)	O2—K^viii^	2.9330 (16)
F—K^iv^	2.6816 (11)	O3—C	1.350 (4)
F—K^v^	2.7389 (11)	O3—K^iii^	2.8477 (17)
O4—K^vi^	2.7599 (17)	O3—K^viii^	2.8477 (17)
O4—H41	0.838 (17)	O3—H3	0.814 (18)
			
F^i^—K—F	82.698 (16)	K^vi^—O4—H41	103.7 (16)
F^i^—K—O1^ii^	117.24 (5)	K—O4—H41	103.7 (16)
F—K—O1^ii^	68.47 (5)	K^vi^—O4—H42	130.3 (11)
F^i^—K—O2	87.60 (4)	K—O4—H42	130.3 (11)
F—K—O2	148.65 (6)	H41—O4—H42	103 (2)
O1^ii^—K—O2	90.17 (5)	C—O1—K^vii^	118.72 (13)
F^i^—K—O4	81.96 (5)	C—O1—K^ii^	118.72 (13)
F—K—O4	135.97 (6)	K^vii^—O1—K^ii^	79.86 (6)
O1^ii^—K—O4	153.18 (6)	C—O2—K^vi^	123.79 (11)
O2—K—O4	71.12 (6)	C—O2—K	123.79 (11)
F^i^—K—O3^iii^	132.40 (6)	K^vi^—O2—K	82.05 (6)
F—K—O3^iii^	80.97 (4)	C—O2—K^iii^	92.42 (14)
O1^ii^—K—O3^iii^	97.43 (4)	K^vi^—O2—K^iii^	141.11 (8)
O2—K—O3^iii^	125.94 (6)	K—O2—K^iii^	89.27 (3)
O4—K—O3^iii^	79.62 (5)	C—O2—K^viii^	92.42 (14)
F^i^—K—O2^iii^	172.55 (4)	K^vi^—O2—K^viii^	89.27 (3)
F—K—O2^iii^	102.32 (3)	K—O2—K^viii^	141.11 (8)
O1^ii^—K—O2^iii^	70.01 (5)	K^iii^—O2—K^viii^	74.13 (5)
O2—K—O2^iii^	90.73 (3)	C—O3—K^iii^	94.04 (14)
O4—K—O2^iii^	90.63 (5)	C—O3—K^viii^	94.04 (14)
O3^iii^—K—O2^iii^	44.48 (6)	K^iii^—O3—K^viii^	76.74 (6)
K^i^—F—K^iv^	84.96 (4)	C—O3—H3	106 (2)
K^i^—F—K^v^	177.20 (5)	K^iii^—O3—H3	136.9 (10)
K^iv^—F—K^v^	97.302 (16)	K^viii^—O3—H3	136.9 (10)
K^i^—F—K	97.302 (16)	O1—C—O2	127.6 (3)
K^iv^—F—K	177.20 (5)	O1—C—O3	117.7 (3)
K^v^—F—K	80.39 (4)	O2—C—O3	114.7 (2)
K^vi^—O4—K	82.01 (6)		

Symmetry codes: (i) −*x*+1, −*y*+1, −*z*; (ii) −*x*+1, −*y*+1, −*z*+1; (iii) −*x*+2, −*y*+1, −*z*+1; (iv) −*x*+1, *y*+1/2, −*z*; (v) *x*, −*y*+3/2, *z*; (vi) *x*, −*y*+1/2, *z*; (vii) −*x*+1, *y*−1/2, −*z*+1; (viii) −*x*+2, *y*−1/2, −*z*+1.

### Hydrogen-bond geometry (Å, º)

**Table:**

*D*—H···*A*	*D*—H	H···*A*	*D*···*A*	*D*—H···*A*
O4—H41···O1^ix^	0.84 (2)	1.89 (2)	2.723 (3)	176 (3)
O4—H42···F^x^	0.84 (2)	1.87 (2)	2.707 (3)	177 (3)
O3—H3···F^ii^	0.81 (2)	1.72 (2)	2.529 (3)	174 (4)

Symmetry codes: (ii) −*x*+1, −*y*+1, −*z*+1; (ix) *x*+1, *y*, *z*; (x) −*x*+2, −*y*+1, −*z*.
